# Smooth-Muscle-Cell-Specific Deletion of CD38 Protects Mice from AngII-Induced Abdominal Aortic Aneurysm through Inhibiting Vascular Remodeling

**DOI:** 10.3390/ijms25084356

**Published:** 2024-04-15

**Authors:** Zhen-Ping Yu, Yi-Kai Wang, Xiao-Yu Wang, Li-Na Gong, Hui-Lan Tan, Mei-Xiu Jiang, Ling-Fang Wang, Guan-Hui Yu, Ke-Yu Deng, Hong-Bo Xin

**Affiliations:** 1 The National Engineering Research Center for Bioengineering Drugs and the Technologies, Institute of Translational Medicine, Nanchang University, Nanchang 330031, China; yuzp0904@163.com (Z.-P.Y.); wyk807431069@126.com (Y.-K.W.); wangxy202201@163.com (X.-Y.W.); linagongg@163.com (L.-N.G.); huilanttan@163.com (H.-L.T.); jiangmxs@163.com (M.-X.J.); wlfang1985@163.com (L.-F.W.); yugh0306@163.com (G.-H.Y.); 2 College of Life Science, Nanchang University, Nanchang 330031, China; 3 School of Pharmacy, Nanchang University, Nanchang 330031, China

**Keywords:** abdominal aortic aneurysm, CD38, sirtuins, vascular smooth muscle cells, phenotypic switching

## Abstract

Abdominal aortic aneurysm (AAA) is a serious vascular disease which is associated with vascular remodeling. CD38 is a main NAD^+^-consuming enzyme in mammals, and our previous results showed that CD38 plays the important roles in many cardiovascular diseases. However, the role of CD38 in AAA has not been explored. Here, we report that smooth-muscle-cell-specific deletion of CD38 (CD38^SKO^) significantly reduced the morbidity of AngII-induced AAA in CD38^SKO^Apoe^−/−^ mice, which was accompanied with a increases in the aortic diameter, medial thickness, collagen deposition, and elastin degradation of aortas. In addition, CD38^SKO^ significantly suppressed the AngII-induced decreases in α-SMA, SM22α, and MYH11 expression; the increase in Vimentin expression in VSMCs; and the increase in VCAM-1 expression in smooth muscle cells and macrophage infiltration. Furthermore, we demonstrated that the role of CD38^SKO^ in attenuating AAA was associated with the activation of sirtuin signaling pathways. Therefore, we concluded that CD38 plays a pivotal role in AngII-induced AAA through promoting vascular remodeling, suggesting that CD38 may serve as a potential therapeutic target for the prevention of AAA.

## 1. Introduction

Cardiovascular diseases are the leading cause of the global mortality and disability, among which aneurysm and aortic dissection are silent killers because more than 95% of the patients are asymptomatic before the rupture occurs [1]. Abdominal aortic aneurysm (AAA), a specific type of aneurysm, is characterized by the progressive dilation of focal abdominal aorta, and the rupture of AAA causes a mortality exceeding 80% [2]. The pathological features of AAA include destruction of elastin, collagen deposition in the media and adventitia, phenotypic switching and apoptosis of smooth muscle cells, infiltration of lymphocytes and macrophages, release of proinflammatory cytokines and proteases, intense oxidative stress, neovascularization, etc. [3,4]. Although numerous molecules and pathways related to AAA have been identified [5], the precise mechanisms of aneurysm have not been not fully elucidated. Currently, surgery is the only effective treatment for repairing rupture or preventing rupture [6]. Therefore, elucidation of the mechanism of AAA formation is favorable to discovering the potential therapeutic targets for the clinical prevention and treatment of AAA.

Vascular smooth muscle cells (VSMCs) are the major cell types of the aorta and play a central role in AAA formation although the sequential pathophysiology is not fully known [7]. Unlike cardiac or skeletal muscle cells, which are terminally differentiated, VSMCs are plastic and capable of phenotypic change in response to local environments [8]. In healthy adult vessels, VSMCs are quiescent with the contractible phenotype. Once vessels are injured by damage stimulation, VSMCs switch to a dedifferentiated phenotype, characterized by increased proliferation, migration, and extracellular matrix synthesis and the decreased expression of contractile markers [9]. Studies have indicated that the mutations of myosin heavy chain 11 (MYH11) and alpha-smooth muscle actin (α-SMA) are able to predict the disruption of VSMC contractile function [10]. Smooth muscle 22α (SM22α) and α-SMA play important roles in maintaining the contractile phenotype of VSMCs and are downregulated in AAA, with elevated SM22α expression being beneficial for preventing AAA formation [11].

CD38 was originally identified as a cell surface marker of human B lymphocytes [12]. CD38 is a multifunctional transmembrane glycoprotein with the catalytic activities of NAD^+^ glycohydrolase, ADPR-ribosyl cyclase, and cADPR hydrolase. CD38 serves as a main NAD^+^-consuming enzyme in mammal cells and a regulator of Ca^2+^ second messengers, including cADPR, NAADP, and ADPRP. NAD^+^ is a central metabolite that influences multiple cellular process in mammalian cells. CD38 influences the activities of sirtuins (SIRTs) such as SIRT1 and SIRT3 via modulating the availability of intracellular NAD^+^ [13,14]. Thus, CD38 is involved in several physiological and pathological conditions through the regulation of tissue NAD^+^ and nicotinamide homeostasis. Our previous studies showed that CD38 deficiency protected against cardiac hypertrophy and ischemia/reperfusion injury, alleviated vascular remodeling in hypertension, attenuated diabetic cardiopathy, and delayed D-galactose-induced cardiomyocyte senescence in mice [15,16,17,18]. In addition, it has been reported that activated Notch signaling was positively correlated with CD38 expression and that inhibitor of notch regressed AngII-induced AAA [19]. Furthermore, the reduction of SIRT1 and SIRT6 in SMC has been associated with vascular senescence and inflammation of AAA [20,21]. All these data suggest that CD38 may participate in AAA formation through reducing the intracellular NAD^+^-mediated suppression of SIRT signaling pathways.

In the current study, we investigated the roles of CD38 in AAA formation with VSMC-specific CD38-knockout (CD38^SKO^Apoe^−/−^) mice. We observed that CD38 deficiency in VSMCs significantly prevented the AngII-induced formation of AAA in Apoe^−/−^ mice, and we ultimately demonstrated that the underlying mechanism of CD38^SKO^ protecting mice from AAA is related to the inhibition of vascular remodeling including the phenotype switch in vascular smooth muscle cells and the macrophage infiltration in vessels.

## 2. Results

### 2.1. Generation and Identification of Smooth-Muscle-Cell-Specific CD38-Knockout Mice

**Figure 1:**
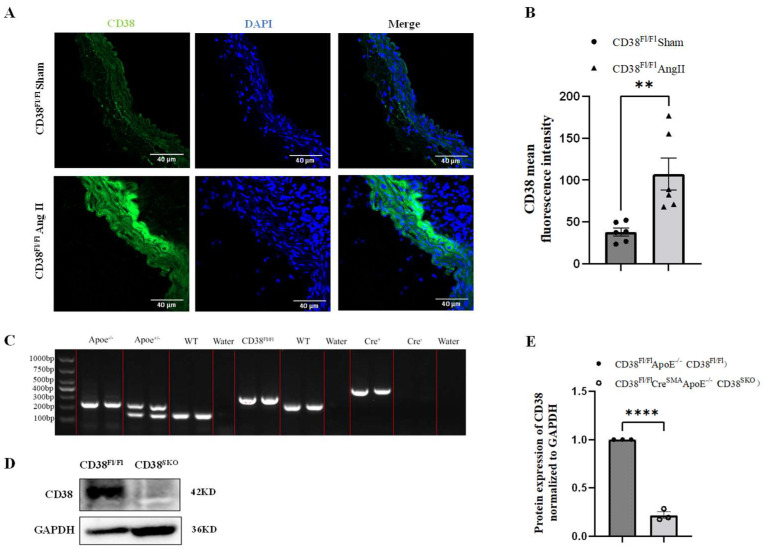
CD38 was upregulated in mice with AngII-induced abdominal aortic aneurysm (AAA) and the generation of smooth-muscle-cell (SMC)-specific deletion of CD38 and conventional deletion of Apoe double-knockout (CD38^SMA^/Apoe^−/−^) mice. (**A**) The images of CD38 expressions were obtained from immunofluorescence analysis of mice with AngII-induced AAA. (**B**) CD38 expression was quantitatively analyzed in (**A**). N = 6; ** *p* < 0.01. (**C**) Apoe alleles, WT, CD38^flx/flx^, and Cre recombinase transgene were validated using PCR, respectively. (**D**) The deletion of CD38 in aortic smooth muscle cells was confirmed using Western blot analysis in CD38^flx/flx^Cre^SMA^Apoe^−/−^ mice. (**E**) Analysis of (**D**). N = 3; **** *p* < 0.0001. Note: CD38^flx/flx^Apoe^−/−^ and CD38^SMA^Apoe^−/−^ are referred to as “CD38^Fl/Fl^“ and “CD38^SMA^”, respectively. Scale bar: 40 μm in (**A**).

To clarify the roles of CD38 in abdominal aortic aneurysm (AAA) formation, mouse AAA models were generated though AngII infusion for 28 days using CD38^Fl/Fl^Apoe^−/−^ mice. The results showed that CD38 expression was significantly upregulated in the AngII-induced AAA model (Figure 1A,B), suggesting that CD38 in vascular smooth muscle cells (VSMCs) might play a role in AngII-induced AAA. To further explore the role of CD38 in aneurysm, smooth-muscle-cell (SMC)-specific CD38 gene knockout (CD38^SKO^) mice were generated by crossing CD38^Fl/Fl^ mice with SMA-Cre mice, in which the exon2 and exon3 of the CD38 gene was flanked by two LoxP sites, and then the CD38^SKO^ mice were mated with Apoe^−/−^ mice to generate the CD38^SKO^Apoe^−/−^ double-knockout mice with a genetic background of C57BL/6. As shown in Figure 1C, the genotypes of WT, CD38^Fl/Fl^, ApoE, and SMA-Cre mice were confirmed via PCR analysis. In addition, the Western blot results showed that the protein levels of CD38 were markedly decreased in the VSMCs of the CD38^SKO^Apoe^−/−^ mice compared with the CD38^Fl/Fl^Apoe^−/−^ mice (Figure 1D,E). These results indicated that the CD38^Fl/Fl^Apoe^−/−^ and CD38^SKO^Apoe^−/−^ mice were successfully generated.

### 2.2. CD38 Deficiency in Smooth Muscle Cells (CD38^SKO^) Protected Mice from AngII-Induced AAA Formation

**Figure 2:**
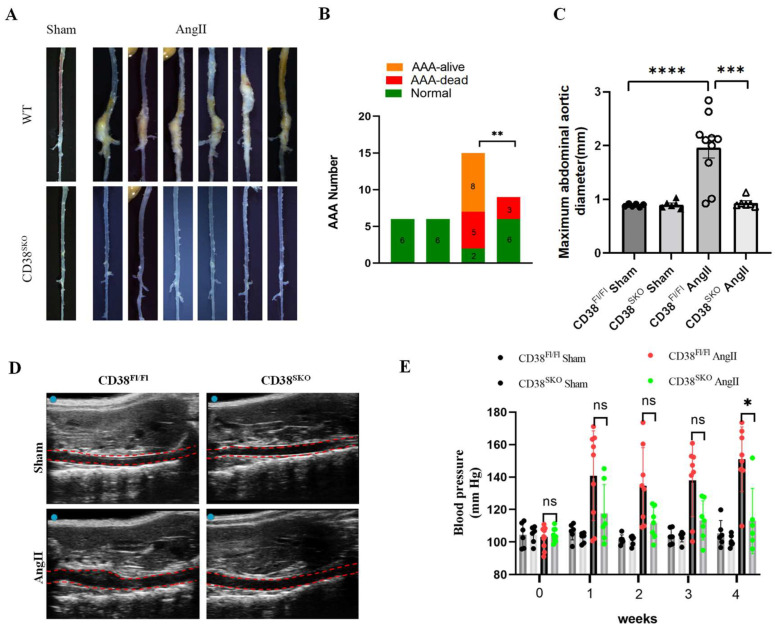
SMC-specific CD38 deficiency (CD38^SKO^) protected against AngII-induced AAA formation in mice. (**A**) Representative images of the entire aortas were obtained from CD38^Fl/Fl^ and CD38^SKO^ mice with or without AngII infusion. (**B**) The incidence of AAA was calculated from CD38^Fl/Fl^ and CD38^SKO^ mice. The difference in the incidence rate was analyzed with the χ^2^ test; ** p < 0.01. (**C**) The maximal abdominal aortic diameters were quantitatively measured for each group; N = 6–10; *** *p* < 0.001, **** *p* < 0.0001. (**D**) The representative images were taken from the ultrasonography of four groups. (**E**) Blood pressures were measured from the four groups after 4-week AngII infusion. N = 6–10; * p < 0.05. Scale bar: 1 cm in (**A**).

Next, the mouse AAA models were generated via AngII infusion for 28 days using CD38^SKO^Apoe^−/−^and CD38^Fl/Fl^Apoe^−/−^ mice. The results showed that SMC-specific deletion (CD38^SKO^) of CD38 significantly reduced the morbidity of AngII-infusion-induced AAA from 86.7% (13/15 mice) of CD38^Fl/Fl^Apoe^−/−^ mice to 33.3% (3/9 mice) of CD38^SKO^Apoe^−/−^ mice; in comparation, the sham operation group had no AAA formation (Figure 2A,B). In addition, the increases of aorta lumen were confirmed with ultrasonographic examination (Figure 2D), and the maximal diameters of the aortas in CD38^Fl/Fl^Apoe^−/−^ mice were remarkably increased after AngII infusion, whereas CD38^SKO^ almost reversed the AngII-infusion-induced increase in maximal aortic diameters in CD38^SKO^Apoe^−/−^ mice compared with CD38^Fl/Fl^Apoe^−/−^ mice (Figure 2C). Furthermore, consistent with our previous study [15], CD38 deficiency reduced the AngII-induced increase in blood pressure (Figure 2E). These results indicated that CD38^SKO^ markedly inhibited AngII-induced AAA formation in mice.

### 2.3. CD38^SKO^ Mitigated Vascular Remodeling in Mouse AAA Models

**Figure 3:**
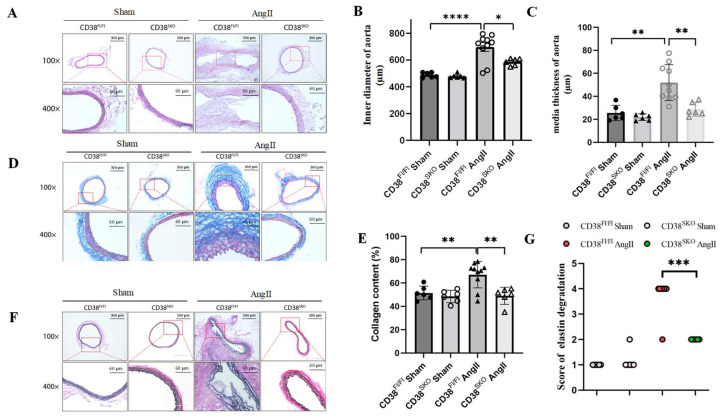
CD38 deficiency in SMCs protected against AngII-induced vascular remodeling in mice. (**A**) Representative images of HE staining. (**B**) Quantification of aortic media thickness. (**C**) Quantification of aortic inner diameter corrected by perimeter. (**D**) Representative images of Masson staining. (**F**) Representative images of EVG staining. (**E**) Quantification of collagen content in (**D**). (**G**) Quantification of elastin degradation grade in (**F**). N = 6–10; * *p* < 0.05; ** *p* < 0.01; *** *p* < 0.001, **** *p* < 0.0001. Scale bar: 300 μm in 100× and 60 μm in 400× of (**A**,**D**,**F**).

To determine the role of CD38 in vascular remodeling, the thickness of the medial layer, the degree of fibrosis, and the integrity of elastic fibers in the aorta were determined via HE, Masson, and EVG staining in AngII-induced mouse AAA models, respectively. HE staining results showed that the aortic diameter and medial thickness were significantly increased in CD38^Fl/Fl^Apoe^−/−^ mice with AngII infusion compared with control mice, whereas CD38^SKO^ almost reversed the AngII-infusion-induced increases in the thickness (Figure 3A,B) and diameter (Figure 3A,C) of the aortas in mice. In addition, Masson staining showed that CD38^SKO^ reduced the AngII-induced increases in the collagen contents of mouse aortas (Figure 3D,E). Furthermore, EVG staining showed that there was obvious elastic fiber fragmentation in AngII-treated CD38^Fl/Fl^Apoe^−/−^ mice, whereas CD38^SKO^ markedly protected against the degradation of the elastic fibers in mouse aortas (Figure 3F,G). These results indicated that CD38^SKO^ might attenuate AngII-induced AAA formation by reducing vascular remodeling.

### 2.4. CD38^SKO^ Attenuated the AngII-Induced Phenotype Switch of SMCs

**Figure 4:**
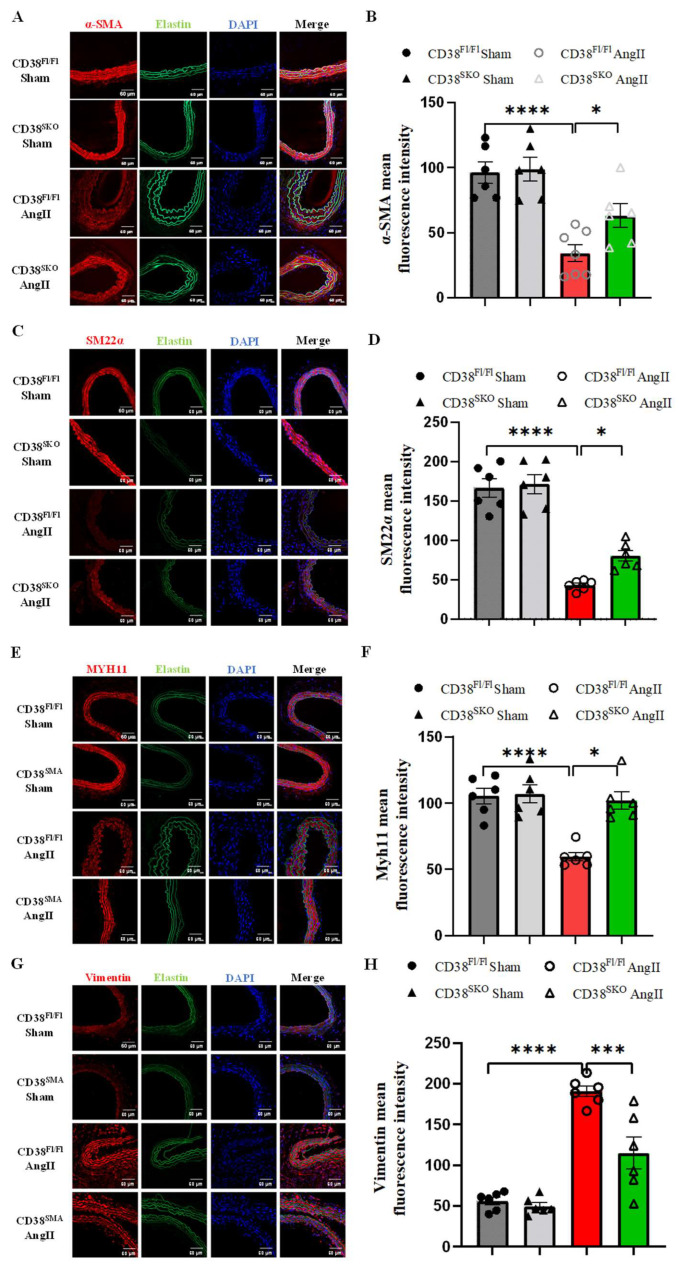
SMC-specific deletion of CD38 inhibited AngII-induced phenotype switch in VSMCs. (**A**) The representative images and quantitative expressions of α-SMA (**A**,**B**), SM22α (**C**,**D**), MYH11 (**E**,**F**), and Vimentin (**G**,**H**) were analyzed via immunofluorescent staining. N = 6; * *p* < 0.05, *** *p* < 0.001, **** *p* < 0.0001. Scale bar: 60 μm in (**A**,**C**,**E**,**G**).

VSMC phenotype switch is closely correlated with vascular remodeling. It has been reported that AngII switches the contractile phenotype to the secretary phenotype in VSMCs to initiate AAA formation. Therefore, we assessed the effect of CD38^SKO^ on the phenotype change of SMCs by conducting immunofluorescence assay. The results showed that CD38^SKO^ prominently prevented the AngII-induced decrease in α-SMA (Figure 4A,B), SM22α (Figure 4C,D), and MYH11 expressions (Figure 4E,F) and the increase in Vimentin expression (Figure 4G,H) in VSMCs of CD38^SKO^Apoe^−/−^ mice compared with control mice, suggesting that CD38^SKO^ reversed the AngII-induced phenotype switch from a differentiated phenotype to a dedifferentiated phenotype in VSMCs.

**Figure 5:**
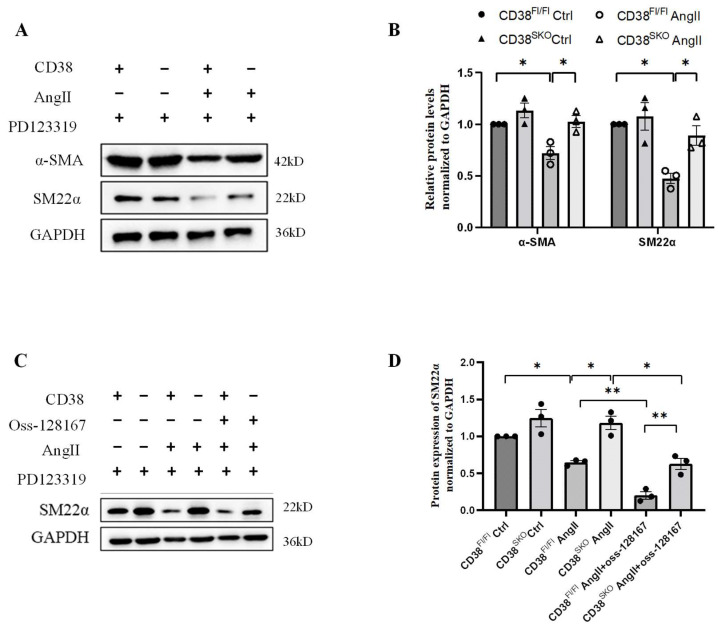
CD38 deficiency in SMCs reversed the AngII-induced decrease in the expression of SMC contractile markers. Primary VSMCs of CD38^Fl/Fl^ and CD38^SKO^ mice were starved for 48 h and pretreated with PD123319 (an AT2R inhibitor) for 2 h and Oss-128167 for 1h, and then AngII was added with or without Oss-128167 (a SIRT 6 inhibitor) for 24 h. (**A**) The protein expressions of α-SMA and SM22α were determined by Western blot under AngII stimulation. (**B**) The quantitative analysis in (**A**). (**C**) The protein expression of SM22α was determined by Western blot under AngII or Oss-128167 treatment. (**D**) The quantitative analysis in (**C**). N = 3; * *p* <0.05, ** *p* <0.01.

To further conform this result, we isolated primary VSMCs to study the effect of CD38^SKO^ on the AngII-induced phenotype switch in vitro. Our results showed that CD38^SKO^ inhibited AngII-induced downregulation of contractile markers such as α-SMA and SM22α in CD38^SKO^Apoe^−/−^ mice compared with CD38^Fl/Fl^Apoe^−/−^ mice (Figure 5A,B), whereas Oss-128167, an SIRT6 inhibitor, partly eliminated the CD38-deficiency-mediated protective role (Figure 5C,D). Taken together, our results indicated that CD38^SKO^ restored the AngII-induced phenotype switch in VSMCs, in which SIRT6 might have contributed to the CD38-deficiency-mediated protective effect against AngII-induced AAA.

### 2.5. CD38^SKO^ Reduced AngII-Induced Macrophage Infiltration and Inflammation in Aortas

**Figure 6:**
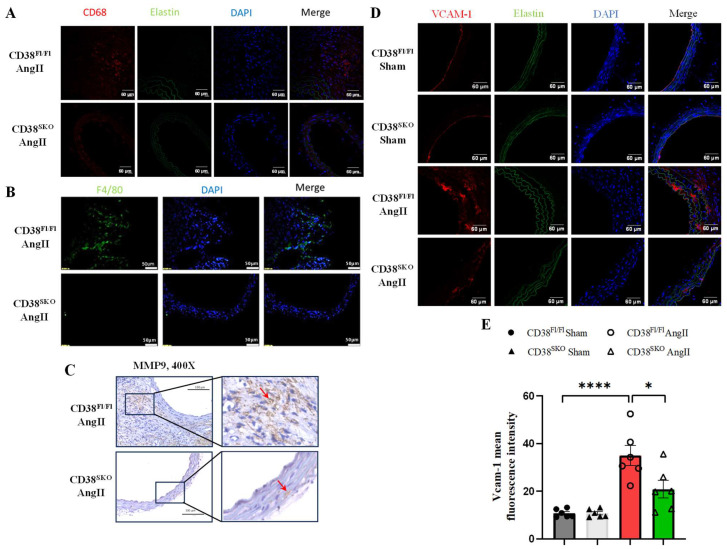

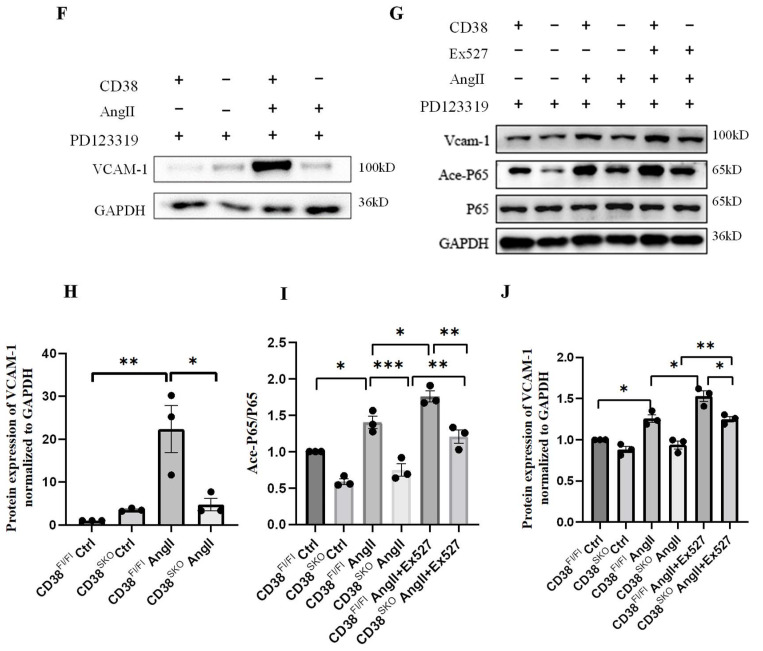
CD38 deficiency in SMCs reduced the AngII-induced infiltration of inflammatory cells in the aortas of CD38^SMA^ mice. The representative images of CD68 (**A**), F4/80 (**B**), and VCAM-1 (**D**) were obtained via immunofluorescent staining in mouse aortas, and the representative images of MMP9 (**C**) were obtained via immunochemical analysis in mouse aortas. MMP9 is shown in brown color pointed by arrow. (**E**) The fluorescent intensities were quantitatively analyzed for VCAM-1. *** *p* < 0.001, * *p* < 0.05. Isolated primary VSMCs were used for (**F**–**J**). Cells were starved for 48 h and pretreated with PD123319 (an AT2R inhibitor) for 2 h and the Ex527 for 1h, then AngII was added with or without Ex527 (a SIRT 1 inhibitor) for 24 h. (**F**) The protein expression of VCAM-1 was determined by Western blot with AngII (1 μM) stimulation. (**G**) The protein expressions of VCAM-1, P65, and acetyl-P65 were determined by Western blot with AngII (1 μM) and Ex527 (10 μM) treatments. (**H**–**J**) The quantitative analysis of the expressions of VCAM-1 (**H**), Acetl-P65/P65 (**I**) with the treatment of Ex527, and VCAM-1 (**J**) with the treatment of Ex527. N = 3; * *p* < 0.05, ** *p* < 0.01, *** *p* < 0.001. Scale bar: 60 μm in (**A**,**D**), 50 μm in (**B**), and 100 μm in (**C**).

It has been reported that AngII promotes the infiltration of the inflammatory cells in the vascular wall, especially in the abdominal aorta aneurysm region, [22] leading to the inflammation in vessels. An earlier study reported an increase in CD68-positive macrophages in the vascular wall in AAA models [23]. Moreover, macrophage-derived MMP9 plays a critical role in AAA formation. Immunofluorescence staining indicated that CD38 deficiency markedly reduced the AngII-induced increase in macrophage marker F4/80-positive cells and M1 macrophage marker CD68-positive cells in the abdominal aortas in CD38^SKO^Apoe^−/−^ mice compared with CD38^Fl/Fl^Apoe^−/−^ mice (Figure 6A,B). Immunohistochemical analysis showed that CD38^SKO^ remarkably reduced the AngII-induced increase in MMP9 level in CD38^SKO^Apoe^−/−^ mice compared with CD38^Fl/Fl^Apoe^−/−^ mice (Figure 6C). It has been reported that activated VSMCs express VCAM-1 and recruit macrophages to lesions [24]. Then, we detected the expression of VCAM-1 in aortic tissues, and the results showed that CD38 deficiency attenuated the AngII-induced increase in the expression of VCAM-1 (Figure 6D,E). Furthermore, we also assessed the effects of CD38 on AngII-induced VCAM-1 expression in mouse primary smooth muscle cells. Our results showed that CD38 deficiency significantly reduced the AngII-induced increase in the expression of VCAM-1 and acetyl-P65 in CD38^SKO^Apoe^−/−^ mice compared with CD38^Fl/Fl^Apoe^−/−^ mice (Figure 6F,H), whereas CD38-deficiency-mediated inhibition was eliminated by the Sirt1 inhibitor Ex527 (Figure 6G–J). These results indicated that CD38^SKO^ inhibited macrophage infiltration through suppressing the Sirt1/NF-κB-signaling-pathway-mediated increase in VCAM-1 expression.

## 3. Discussion

Abdominal aortic aneurysm (AAA) accounts for about 95% of aneurysms, which refers to the decrease of local tension of the vascular wall due to the decreased local elasticity of the vascular wall, leading to aneurysm rupture in severe cases. AAA is involved in vascular remodeling including the phenotype switch and apoptosis of smooth muscle cells, the degradation of the extracellular matrix (ECM), and the infiltration of inflammatory cells in aortas. CD38 is a multifunctional enzyme with the catalytic activities of NAD^+^ glycohydrolase, ADPR-ribosyl cyclase, and cADPR hydrolase. Importantly, CD38 serves as a main NAD^+^-consuming enzyme that plays a key role in the regulation of intracellular NAD^+^ levels, in which NAD^+^ is a central metabolite that influences multiple cellular process in mammal cells. In our previous studies, we found that CD38 deficiency protected against cardiac hypertrophy and ischemia/reperfusion injury, alleviated vascular remodeling in hypertension, attenuated diabetic cardiopathy, and delayed D-galactose-induced cardiomyocyte senescence in mice [15,16,17,18]. In the present study, we observed that CD38 deficiency in smooth muscle cells significantly inhibited AngII-induced AAA in CD38^SKO^Apoe^−/−^ mice, which led to a remarkable reduction of morbidity through decreasing the aortic diameter and medial thickness, suppressing deposition of the collagens, and preventing the degradation of elastic fibers. Furthermore, we demonstrated that the protectory effect of CD38 deficiency on AngII-induced AAA was associated with the alleviation of vascular remodeling including the inhibition of the phenotype switch and the reduction of the infiltration of inflammatory cells, suggesting that CD38 may be a potential therapeutic target for the prevention and treatment of AAA formation.

Vascular remodeling is a typical feature of AAA, which is characterized by the increase of the thickness of media and the enlargement of luminal diameter [25]. The expansion of aneurysms is directly related to the decrease of tension strength in collagen fibers and elastic fibers, so pathological vascular remodeling is one of the underlying causes of AAA. In the present study, we found that CD38 deficiency in SMCs markedly inhibited the AngII-induced increase in lumen size and medial thickness, collagen deposition, and the elastin degradation, suggesting that the protection of CD38 deficiency on AngII-induced AAA formation is associated with the inhibition of vascular remodeling.

Phenotype switch in VSMCs is closely correlated with many vascular diseases, [26] which has been considered to be a key mechanism in vascular remodeling and aneurysm [27]. Smooth muscle cells in the vascular wall mainly consist of contractile phenotype cells, which allow the vessel to maintain normal contraction and relaxation. The phenotype switch of VSMCs is a first step during the progression of aortic aneurysms [28]. Therefore, inhibiting the phenotype switch in smooth muscle cells may be a potential therapeutic strategy for the prevention and treatment of aortic aneurysms or aortic dissections [29]. SM22α, α-SMA, and MYH11 are the hallmarks of SMCs and are usually downregulated in the condition of AAA [30]. In the current study, CD38 deficiency in SMCs significantly reversed the AngII-induced decreases in the expression of contractile marker proteins such as α-SMA, SM22α, and MYH11 and the upregulation of the expression of the synthetic marker protein Vimentin in the aorta of the AAA animal model, indicating that CD38 participated in the phenotype switch from a contractile phenotype to a synthetic phenotype. Furthermore, we observed that Oss-128167, an SIRT6 inhibitor, partially eliminated the protective role of CD38^SKO^ in the phenotype switch of AngII-mediated AAA, suggesting that the CD38-deficiency-mediated protective effects in AngII-induced AAA may be related to the NAD^+^/Sirt6 signaling pathway.

An accumulation of inflammatory cells in aortas is one of the pathological features of AAA. VCAM-1 plays an essential role in promoting the adhesion and transmigration of inflammatory cells to the injured site, in which the protein is upregulated in pathological conditions and the activated VSMCs. Under physiological conditions, VSMCs are absent of VCAM-1 expression, but studies indicate that there is an increase in VCAM-1 expression in VSMCs during the phenotype changes [24]. What is more, AngII is an inducer of VCAM-1 expression in VSMCs [31]. In a vascular injury model, single-cell RNA-seq analysis showed that the inflammatory cells mainly cross-talked with smooth muscle cells rather than other cells including endothelial cells [32], which supported our results indicating that the upregulation of VCAM-1 expression in VSMCs plays an important role in vascular wall inflammation. Our data showed that CD38^SKO^ significantly reduced the AngII-induced increases in the expression of VCAM-1 and P65 acetylation which were eliminated by Ex527, a SIRT1 inhibitor. Macrophage-derived MMP9 is an important cause of the degradation of elastin fibers. In accordance with inhibition of VCAM-1 expression, there was less inflammatory cell infiltration and lower MMP9 expression in the aortas of CD38^SKO^Apoe^−/−^ mice. These results indicated that CD38 elevated the infiltration of inflammatory cells through inhibiting the Sirt1-mediated NFκB pathway. Given that AngII-mediated cardiovascular disease is mainly achieved by acting on AT1R in a pathological condition and that the in vitro culture of VSMC-mediated injury is related with the expression of AT2R [33,34], PD123319 (an AT2R inhibitor) was used in our in vitro experiments to eliminate the influence of AT2R.

NAD^+^ serves as a crucial metabolic cofactor required for the activity of multifunctional enzymes such as sirtuins. CD38 is a main enzyme for NAD^+^ degradation in mammals, and mice with CD38 deficiency were reported to have a 10- to 20-fold increases in intracellular NAD^+^ level compared with wild-type mice [35]. CD38 deficiency was able to exert multiple effects on cytoplasm, nuclei, and mitochondria due to the diversity of sirtuins in their distributions and functions. Growing evidence from animal models suggests that NAD^+^-boosting therapy is beneficial to AAA treatment. For example, the reduced content of nicotinamide phosphoribosyltransferase (NAMPT) was observed in patients with dilated aortopathy [36], and VSMC *nampt*-deficiency in mice was found to aggravate AngII-induced aortic dissection due to the decrease of the intrinsic NAD^+^ levels [36]. Niacin, a precursor of NAD^+^, was shown to protect against both AngII- and CaCl_2_-induced AAA by restoring the NAD^+^ level and increasing SIRT1 activity in the aorta [37]. Studies have shown that SIRT1 and SIRT6 reduces abdominal or thoracic aortic aneurysms by reducing the senescence and inflammation in VSMCs [20,21,38]. In the present study, the CD3- deficiency-mediated protective effects against AngII-induced AAA were partially attenuated by SIRT1 and SIRT6 inhibitors, respectively, suggesting that the NAD^+^/SIRT1 and NAD^+^/SIRT6 signaling pathways may play a role in AngII-induced AAA formation.

In conclusion, our data demonstrated that CD38 deficiency in SMCs protects against AngII-induced AAA formation by attenuating vascular remodeling including the inhibitions of SMC phenotype switch and macrophage infiltration in aortas, suggesting that CD38 may be a potential therapeutic target for AAA formation.

## 4. Materials and Methods

### 4.1. Experimental Animals

Animal experimental protocols were approved by the Transgenic Animal Center of Institute of Translational Medicine, Nanchang University. Apoe^−/−^ mice were purchased from Beijing Vital River Laboratory Animal Technology Co., Ltd. (Beijing, China). CD38^Fl/Fl^ mice were generated by Cyagen Biosciences Inc. (Suzhou, China). SMA-Cre mice were granted from the Collaborative Innovation Center of Model Animal, Wuhan University. All the mice mentioned above had the C57BL/6 genetic background. Considering that AAA incidence is about 4.8-fold higher in men than in women [39], we chose three—to-four-month-old male mice in our study. All mice in this study were fed with sterilized normal mouse chow and housed in a specific pathogen-free (SPF) laboratory animal room with a 12-h dark/light cycle under a suitable temperature and humidity.

### 4.2. Cell Culture

Mouse primary vascular smooth muscle cells (mVSMCs) were isolated from age-matched mice using an enzymatic dissociation method. In brief, thoracic aorta and abdominal aorta were isolated, and the surrounding fat tissue was removed. Aortas were rinsed with Hanks’ Balanced Salt Solution (HBSS) to remove blood clots and impurity. Aortas were incubated with 0.1% type II collagenase at 37 °C in a cell incubator for 10 min. The adventitia were removed, and endothelial cells were wiped off carefully to obtain a relatively pure media. Then, the cells were incubated with media containing 0.1% type II collagenase in a cell incubator again until formation of a mixture of few cell clumps and single cells. After termination and resuspension, the cells were cultured in primary aortic smooth muscle cell medium according to the directions of the ATCC at 37 °C in a humidified atmosphere of 95% air and 5% CO_2_. For the experiment, mVSMCs were used at 3–5 passages to maintain good conditions. Before experiments, mVSMCs were starved for 48 h in serum-free DMEM and treated with AngII for 24 h. The AT2R inhibitor PD123319 (10 μM) was added to mVSMCs 2 h before AngII stimulation to diminish the influence of the in vitro culturing of VSMCs in the AngII-treated model and to acquire more exact results. SIRT1 inhibitor Ex527 (10 μM) and SIRT6 inhibitor Oss-128167 (100 μM) were added 1 h before AngII stimulation.

### 4.3. AngII-Induced AAA Model

Three to four month-old male CD38^Fl/Fl^Apoe^−/−^ and CD38^SKO^Apoe^−/−^ mice with a C57BL/6 genetic background were used for experiments. Mice were anesthetized with isoflurane vaporizer, and then an incision was made on the super back for the placement of osmotic mini-pumps (Model ALZET 2004; Durect Corp, Cupertino, CA, USA). AngII (MB1677; Dalian Meilun Biotech Co., Ltd., Dalian, China) was infused into CD38^SKO^Apoe^−/−^ or CD38^Fl/Fl^Apoe^−/−^ mice at 1000 ng/kg/min via osmotic mini-pumps. At 4 weeks after AAA induction, the mice were euthanized, and whole aortas were collected. AAA was defined as a more than 50% enlargement of the maximal diameter of the aorta.

### 4.4. Blood Pressure Detection and Aortic Aneurysm Ultrasonography

Mouse blood pressure was measured with the noninvasive tail-cuff method once a week. After 4 weeks of AngII infusion, the surviving mice were anesthetized with an isoflurane anesthesia machine and underwent ultrasound imaging with MX400 probe on the VisualSonics Vevo2100 System (Fujifilm VisualSonics, Inc., Toronto, Canada).

### 4.5. Morphological and Histological Examination

The mice were anesthetized, and the aorta from the ascending aorta along the heart to the iliac arteries was isolated. The photography of whole aortas was immediately completed with a stereo microscope with a agarose gel plate, and the external diameter of the aorta was measured at the most enlarged position with vernier calipers. Then, the aneurysm region or the equivalent position of non-aneurysm from the aorta segment of the mice was acquired. Aortic tissues were embedded with OCT compound and frozen in liquid nitrogen and then stored at −80 °C prior to sectioning. Subsequently, 5 μm thick frozen sections were prepared from OCT-embedded aortic tissues for hematoxylin and eosin (H&E) staining, Masson staining, and Elastica van Gieson (EVG) staining under standard protocols. Sections were fixed with 4% paraformaldehyde (PFA) before staining. Considering the influence of the aortic lumen shape, we calculated the inside diameter using the circumference. The thickness of the medial layer and circumference was measured using the built-in software of the laser scanning confocal microscope.

### 4.6. Immunohistochemistry and Immunofluorescence

Aortic frozen sections were removed from -80 freezer and fixed for 10 min in 4% PFA at room temperature. Then, the sections were washed for 10 min in PBS 3 times. Non-specific binding sites were blocked with 5% goat serum (in PBS) for 1 h at room temperature. The primary antibodies were diluted with 2% BSA and incubated with tissues sections overnight at 4 °C. The primary antibodies were removed, and the sections were washed with PBST for 3 times next day. For immunohistochemistry, sections were incubated with corresponding horseradish-peroxidase-(HRP)-labeled secondary antibodies (in 2% BSA) and washed for 10 min with PBST 3 times. Color development was performed using a DAB color development kit. For immunofluorescence, fluorescent secondary antibodies were used instead of HRP-conjugated secondary antibodies and did not need DAB color development. DAPI was used to label nuclei, and elastic fiber autofluorescence was excited with a 488-nm laser.

### 4.7. Western Blot

At the endpoint of treatment, cultured cells were lysed with RIPA buffer containing protease inhibitor cocktail and phosphatase inhibitor. The supernatant was extracted for the measurement of total protein with a protein assay kit (BCA). Samples were dissociated with SDS-PAGE and then transferred to PVDF membranes and incubated with primary antibody for GAPDH [Kangchen Biotech (Shanghai, China), Cat# KC-5G4, RRID:AB_2493106], α-SMA [Cell Signaling Technology (Beverly, MA, USA), Cat# 19245, RRID:AB_2734735], SM22α (Cell Signaling Technology, Cat# 52011), Vimentin [Solarbio (Beijing, China), Cat# GB11192, RRID:AB_2814685], P65 Cell Signaling Technology Cat# 8242S), acetyal-P65 (Cell Signaling Technology Cat# 12629S), and VCAM-1 (Cell Signaling Technology Cat# 39036, RRID:AB_2799146) at 4 °C overnight. The membranes were washed 3 times and incubated for 1 h at room temperature with horseradish-peroxidase-(HRP)-conjugated secondary antibodies. Images were quantified using the digital gel image analysis system TANON 5500, and ImageJ Version 1.46r analysis software was used for protein expression analysis.

### 4.8. Statistical Analysis

All statistical analyses were performed using Graphpad Version 9.3.1 Software (Graphpad Software, Inc., La Jolla, CA, USA). Results are expressed as the mean ± SEM unless otherwise noted. Differences between two groups were analyzed with the Student’s *t*-test, and differences between multiple groups were analyzed with one-way ANOVA. The incidence rate was analyzed with the χ^2^ test. The score of elastin degradation was analyzed with the Mann–Whitney U method. A *p* value less than 0.05 was considered to be significant.

## Data Availability

Data is contained within the article.
